# Epidemiology of Imported Infectious Diseases, China, 2005–2016

**DOI:** 10.3201/eid2501.180178

**Published:** 2019-01

**Authors:** Yali Wang, Xuan Wang, Xiaobo Liu, Ruiqi Ren, Lei Zhou, Chao Li, Wenxiao Tu, Daxin Ni, Qun Li, Zijian Feng, Yanping Zhang

**Affiliations:** Chinese Center for Disease Control and Prevention, Beijing, China (Y. Wang, X. Liu, R. Ren, L. Zhou, C. Li, W. Tu, D. Ni, Q. Li, Z. Feng, Y. Zhang);; National Institute for Communicable Disease Control and Prevention, Chinese Center for Disease Control and Prevention, Beijing (X. Liu);; 302 Military Hospital of China, Beijing (X. Wang)

**Keywords:** communicable diseases, epidemiology, prevention and control, China, vector-borne infections, viruses, imported infectious disease, malaria, MERS, dengue, chikungunya, yellow fever, acute flaccid paralysis, poliomyelitis, influenza, Zika

## Abstract

Imported infectious diseases are becoming a serious public health threat in China. However, limited information concerning the epidemiologic characteristics of imported infectious diseases is available. In this study, we collected data related to imported infectious diseases in mainland China from the National Information Reporting System of Infectious Diseases and analyzed demographic, temporal, and spatial distributions. The number of types of imported infectious diseases reported increased from 2 in 2005 to 11 in 2016. A total of 31,740 cases of infectious disease were imported to mainland China during 2005–2016; most of them were found in Yunnan Province. The cases were imported mainly from Africa and Asia. As a key and effective measure, pretravel education should be strengthened for all migrant workers and tourists in China, and border screening, cross-border international cooperation, and early warning should be further improved.

The advance of globalization, frequent personnel exchanges, and close international trade cooperation make it possible for infectious diseases from all over the world to be imported into China. The number of persons entering and leaving China has been steadily increasing, from 302 million person-times in 2005 to 570 million person-times in 2016, and the number of visitor arrivals in Yunnan Province has increased from 1.5 million (1.0 million foreigners) in 2005 to 6.0 million (4.5 million foreigners) in 2016 (*1*). According to the China Statistical Yearbook (http://www.stats.gov.cn/english/Statisticaldata/AnnualData), the number of inbound tourists from Africa has grown from 238,000 in 2005 to 589,000 in 2016, whereas the number of inbound tourists from Asia has grown from 12.5 million in 2005 to 18.0 million in 2016. The number of migrant workers leaving China rose in the same period, from 272,900 to 494,200. Some infectious diseases have been reemerging, such as epidemic hemorrhagic fever and malaria, which previously had basically been controlled, and wild poliovirus, which had been eliminated in China in 2000. After >10 years without a case of wild poliovirus infection in China, an outbreak of wild poliovirus infection occurred in 2011 in Xinjiang Uyghur Autonomous Region (*2*). A few emerging infectious diseases, such as Middle East respiratory syndrome (MERS), yellow fever, and Zika virus disease, have also been imported into China.

Since 2010, annual numbers of autochthonous malaria cases in China have fallen to unprecedentedly low levels; only hundreds of cases now occur in limited areas (*3**–**5*), whereas the number of imported cases has risen substantially (*6*). Malaria is a mosquitoborne infectious disease that caused a heavy health and economic burden in the past. *Anopheles sinensis, An. lesteri*, *An. minimus*, and *An. dirus* mosquitoes are considered to be 4 major malaria vectors in China. As a main vector of transmitting malaria, *An. sinensis* mosquitoes are present in all provinces and regions except Qinghai and Xinjiang; *An. lesteri* mosquitoes are found in areas south of 34°N latitude, *An. minimus* mosquitoes in mountainous and hilly areas south of 33°N latitude, and *An. dirus* mosquitoes mainly in the tropical jungles of Hainan. In China, *Aedes albopictus* and *Ae. aegypti* mosquitoes are 2 major vectors that can transmit flaviviruses and alphaviruses, which cause diseases such as dengue fever, chikungunya, and yellow fever. According to a recent investigation by the Chinese Center for Disease Control and Prevention (CDC), *Ae. aegypti* mosquitoes are found in 10 cities or counties in Yunnan Province, 7 cities or counties in Hainan Province, 1 city in Guangdong Province, and areas south of the Tropic of Cancer in Taiwan. *Ae. albopictus* mosquitoes are widely present in 25 provinces in China, in the southeastern parts of a line between Shenyang in Liaoning Province and Motuo County in Tibet (*7**,**8*).

This study describes the epidemiologic characteristics of imported infectious disease cases during 2005–2016 in mainland China. It also provides scientific information for prevention and control in the future.

## Materials and Methods

### Surveillance System

In China, since the establishment in the 1950s of the Notifiable Disease Reporting System (NDRS), which was the main communicable disease surveillance system, many disease-specific surveillance systems have been developed as complements to NDRS. In 2004, the National Information Reporting System of Infectious Diseases, an internet-based real-time information reporting technique, was integrated into NDRS to improve case-based reporting. The workflow includes data collection, data management, data use, and report dissemination. Once a case of notifiable infectious disease or emerging infectious disease is identified, it is mandatory for doctors in all hospitals and clinics to report to the network within the statutory period. CDCs at various levels (e.g., provincial) are in charge of data checking, utilization, and timely information feedback by uploading the reports on the websites. All the hospitals and CDCs at different levels can download these reports and know the national status in a timely manner, which makes the epidemic information transparent. The completeness and timeliness of case reporting have improved dramatically since 2004 (*9*). Therefore, this study takes 2005 as the first year of analysis, considering data quality and stability.

Once an emerging infectious disease is found that is not yet included in statutory reporting but is of considerable interest, the National Health Commission of the People’s Republic of China usually organizes relevant experts in clinical, epidemiologic, etiologic, and other areas to assess its risk of becoming epidemic, spreading range, influence extent, and social burden to determine whether the disease should be added to the list of notifiable infectious diseases. The results are submitted to the National People’s Congress for deliberation and adoption. At the same time, the emerging infectious disease is required to be reported under “other categories of infectious diseases” in the National Information Reporting System of Infectious Diseases.

### Data Collection

Using data from the National Information Reporting System of Infectious Diseases (which does not include Hong Kong, Macao, and Taiwan), we performed a retrospective analysis of imported infectious diseases in mainland China during January 1, 2005–December 31, 2016. We selected data according to reporting date, reporting area, and final confirmation. The cases reported in the National Information Reporting System of Infectious Diseases, including laboratory-confirmed cases and clinically diagnosed cases, were diagnosed by clinicians according to the unified national diagnostic criteria (*10*).

### Population and Case Definition

We divided the population into citizens of China and foreign citizens according to their native countries, so the imported cases included not only citizens of China returning from migrant work or other travel abroad but also foreign visitors, expatriates, and migrant workers in China. The major occupational categories of patients were migrant workers, doctors, teachers, students, and retirees.

Local CDC staff ascertained a potential imported case according to the field investigation results after diagnosis. A case in a patient who had visited or lived in an endemic or epidemic area outside China within the longest incubation period before the date of onset was classified as an imported case. Conversely, a case would be classified as a domestic case if there was no evidence of an infection acquired abroad.

### Statistical Analysis

We entered and managed the data using Microsoft Excel 2010 (https://office.microsoft.com/excel). We used SPSS 18.0 (https://www.ibm.com/analytics/spss-statistics-software) to describe characteristics of imported infectious diseases regarding geographic and temporal distribution, gender, age, region, or country of origin. We created distribution maps of imported cases using ArcGIS 10.3 (http://www.arcgis.com/).

## Results

During 2005–2016, a total of 31,740 cases of infectious diseases were imported into China. These included 27,497 cases of malaria, 3,351 cases of dengue fever, 773 cases of influenza A(H1N1), 24 cases of Zika virus disease, 18 cases of chikungunya fever, 11 cases of yellow fever, 2 cases of acute flaccid paralysis caused by poliomyelitis, 1 case of MERS, and 65 cases of other infectious diseases (Table 1). The study revealed that Yunnan Province witnessed the largest number of imported cases of infectious diseases during the study period.

**Table 1:** Imported infectious diseases in mainland China, 2005–2016*

Disease	Indigenous cases	Imported cases	Total cases	Imported cases, %	Main reporting provinces/cities	Main location(s) of acquisition
Malaria						
* Plasmodium vivax*	149,675	10,506	160,181	6.6	Yunnan	Asia (Myanmar)
* P. falciparum*	8,080	14,896	22,976	64.8	Yunnan, Guangxi	Asia (Myanmar), Africa (Ghana)
Undetermined species	29,929	2,095	32,024	6.5	NA	NA
Subtotal	187,684	27,497	215,181	8.1	NA	NA
Dengue fever	58,602	3,351	61,953	5.4	Yunnan	Asia (Myanmar)
Influenza A(H1N1)	75,924	773	76,697	1.0	Beijing, Guangdong	Asia, USA
Chikungunya	244	18	262	6.9	Guangdong, Zhejiang	Angola, Philippines
Lyme disease	162	2	164	1.2	Beijing	Germany
EHF	44,682	7	44,689	0.0	NA	NA
Scrub typhus	68,841	7	68,848	0.0	Hubei	Thailand
VL	1,317	14	1,331	1.1	Hubei, Sichuan	Afghanistan, Spain
JE	5,656	17	5,673	0.3	NA	NA
AFP	27,056	2	27,058	0.0	NA	NA
Loiasis	0	11	11	100.0	Beijing, Shandong, Hubei	Cameroon, Congo, Gabon
EHEC	4	3	7	42.9	Jiangsu	Thailand
CCHF	0	1	1	100.0	Beijing	Congo
Schistosomiasis	0	1	1	100.0	Beijing	Nigeria
MERS	0	1	1	100.0	Guangdong	South Korea
Yellow fever	0	11	11	100.0	Fujian	Africa (Angola)
Zika virus	0	24	24	100.0	Guangdong	South America (Venezuela)
Total	470,172	31,740	501,911	6.3		

*AFP, acute flaccid paralysis; CCHF, Crimean-Congo hemorrhagic fever; EHEC, *Escherichia coli* O157:H7; EHF, epidemic hemorrhagic fever; JE, Japanese encephalitis; MERS, Middle East respiratory syndrome; NA, not applicable; VL, visceral leishmaniasis

### Epidemiologic Profile of Imported Cases in Mainland China, 2005–2016

#### Demographic Characteristics of Imported Cases

**Figure 1:**
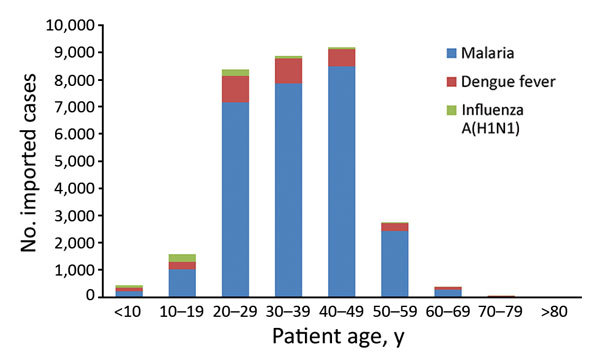
Distribution by age group of imported malaria, dengue fever, and influenza A(H1N1) cases in mainland China, 2005–2016.

Most persons with imported cases were male. The median age of patients with imported cases of malaria was 39 years; of patients with dengue fever, 32 years; and of influenza A(H1N1), 20 years (Table 2; Figure 1). Among these imported cases, 2,470 were reported in foreigners and 29,270 in travelers from China; 18,932 cases were reported in migrant workers from China and 12,808 in persons with other occupations. Migrant workers from China accounted for 65.2% of imported malaria cases and 28.1% of imported dengue fever cases.

**Table 2:** Demographic characteristics of persons with imported cases of infectious disease in mainland China, 2005–2016*

Disease	Sex, no. (%)		Mean age, y (range)	Native country, no. (%)			Occupation, no. (%)		Total
M	F	China	Other	Migrant worker	Other
Malaria	25,819 (93.9)	1,678 (6.1)	39 (1–83)	26,048 (94.7)	1,449 (5.3)		17,929 (65.2)	9,568 (34.8)	27,497
Dengue fever	2,138 (63.8)	1,213 (36.2)	32 (1–86)	2,403 (71.7)	948 (28.3)		943 (28.1)	2,408 (71.9)	3,351
Influenza A(H1N1)	758 (98.1)	15 (1.9)	20 (0.7–75)	731 (94.6)	42 (5.4)		15 (1.9)	758 (98.1)	773
Chikungunya	15 (83.3)	3 (16.7)	25 (20–47)	15 (83.3)	3 (16.7)		4 (22.2)	14 (77.8)	18
Lyme disease	1	1	NA	1	1		0	2	2
Scrub typhus	3 (42.9)	4 (57.1)	23 (3–42)	2 (28.6)	5 (71.4)		0	7	7
VL	13 (92.9)	1 (7.1)	34 (25–49)	14 (100.0)	0 (0.0)		13 (92.9)	1 (7.1)	14
JE	12 (70.6)	5 (29.4)	8 (1–54)	5 (29.4)	12 (70.6)		0	17	17
EHF	7 (100.0)	0	48 (25–71)	6 (85.7)	1 (14.3)		5 (71.4)	2 (28.6)	7
AFP	0	2	NA	2	0		0	2	2
Loiasis	10 (90.9)	1 (9.1)	37 (22–60)	11 (100.0)	0 (0.0)		8 (72.7)	3 (27.3)	11
EHEC	3	0	63 (43–74)	3	0		0	3	3
CCHF	1	0	35	1	0		0	1	1
Schistosomiasis	1	0	28	1	0		0	1	1
MERS	1	0	43	0	1		0	1	1
Yellow fever	8 (72.7)	3 (27.3)	42 (18–53)	11 (100.0)	0 (0.0)		8 (72.7)	3 (27.3)	11
Zika virus	15 (62.5)	9 (37.5)	30 (5–55)	16 (66.7)	8 (33.3)		7 (29.2)	17 (70.8)	24
Total	28,805 (90.8)	2,935 (9.2)	NA	29,270 (92.2)	2,470 (7.8)		18,932 (59.6)	12,808 (40.4)	31,740

*AFP, acute flaccid paralysis; CCHF, Crimean-Congo hemorrhagic fever; EHEC, *Escherichia coli* O157:H7; EHF, epidemic hemorrhagic fever; JE, Japanese encephalitis; MERS, Middle East respiratory syndrome; NA, not applicable; VL, visceral leishmaniasis

#### Trends in Imported Cases in Mainland China, 2005–2016

In 2005, only 2 types of imported infectious diseases were reported in mainland China: malaria and dengue fever. Chikungunya was imported into China in 2008 and influenza A(H1N1) in 2009; Lyme disease has been imported since 2012. Since 2013, the number of types of imported infectious diseases reported has increased each year. A total of 6 types of diseases, including scrub typhus, visceral leishmaniasis (VL), Japanese encephalitis (JE), epidemic hemorrhagic fever, loiasis, and Crimean-Congo hemorrhagic fever, started to be imported in 2013. Imported cases of *Escherichia coli* O157:H7 infection, schistosomiasis, and MERS were reported in 2015. In 2016, 24 cases of Zika and 11 cases of yellow fever were imported. The types of reported imported infectious diseases increased dramatically after 2013 and reached 11 types in 2016, compared with only 2 types in 2005.

**Figure 2:**
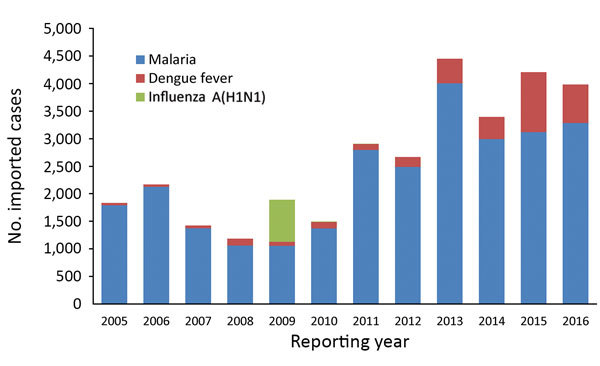
Annual number of imported malaria, dengue fever, and influenza A(H1N1) cases in mainland China, 2005–2016.

We found that imported cases of malaria showed an upward trend from 2005 to 2016. The imported cases of dengue fever increased year by year; increased sharply in 2013 and 2015, with a peak in 2015 (1,094 cases); and declined slightly in 2016 (Figure 2).

#### Seasonal Distribution of Imported Cases

**Figure 3:**
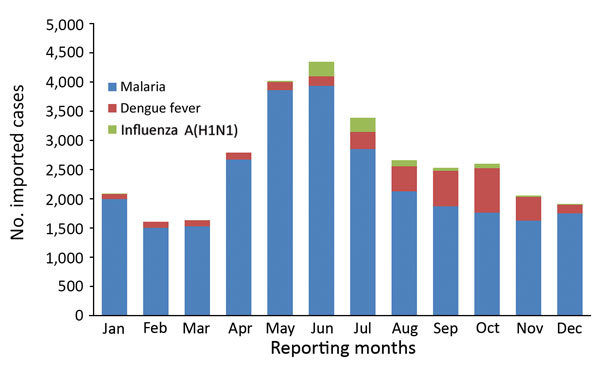
Monthly distribution of imported malaria, dengue fever, and influenza A(H1N1) cases in mainland China, 2005–2016.

Many of the main imported diseases in mainland China exhibited seasonality. Most yellow fever cases were imported in March. There was usually a higher incidence of imported malaria during April–August, reaching a peak in May and June. All the JE cases were imported during June–September, whereas incidence of imported dengue fever usually peaked in October (Figure 3). In particular, the cases of Zika were imported mainly through international airports around major festival events, such as the Spring Festival (celebrating the lunar new year), May Day, the Mid-Autumn Festival, and the National Day (October 1) (*11*).

### Spatial Distribution of Imported Diseases

#### Provinces with Imported Diseases

**Figure 4:**
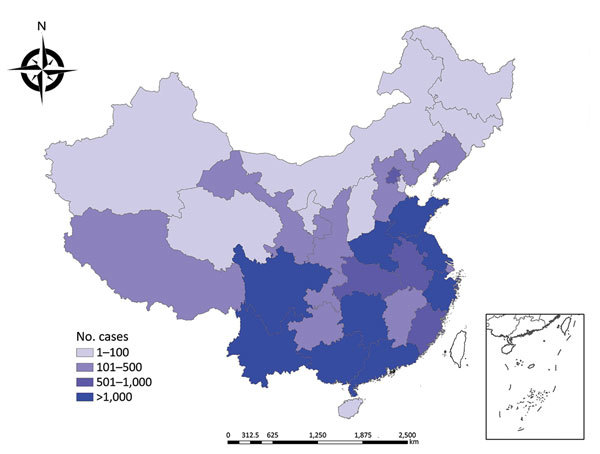
Number of cases of imported infectious diseases in mainland China, by province, 2005–2016.

All 31 provinces across the country reported imported malaria (Figure 4); 36.1% (9,931 cases) were reported in Yunnan Province. The number of imported malaria cases in Yunnan Province was generally decreasing, however, whereas it had been slowly increasing in Sichuan, Henan, Jiangsu, and Zhejiang Provinces and other southeastern provinces year by year. In 2013, imported malaria increased sharply in Guangxi Province, which had the largest number of imported malaria cases in China for that year (1,261/4,067; 31.0%), accounting for 52.7% (1,261/2,394) of the total imported malaria cases in this province during 2005–2016 (data not shown).

Twenty-seven provinces across the country, all except Shanxi, Qinghai, Ningxia, and Tibet, reported dengue fever during 2005–2016. Among all imported dengue fever cases, 42.9% (1,439/3,351) were reported in Yunnan Province (data not shown). Influenza A(H1N1) was imported mainly into Beijing, Guangdong, and other major port cities (data not shown).

#### Origin Region/Country of Imported Diseases

**Figure 5:**
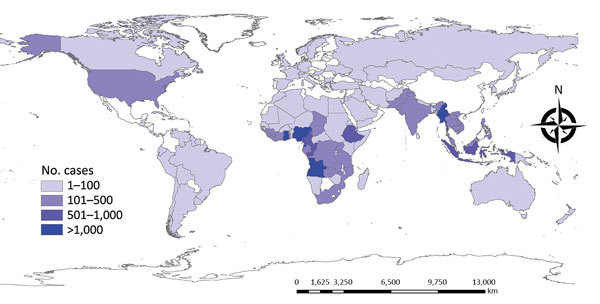
Number of cases of infectious diseases exported to mainland China, by country of origin, 2005–2016.

According to our analysis, Africa and Asia were the main regions of origin of imported cases; 15,021 (47.3%) patients came from Africa and 12,581 (39.6%) from Asia (Figure 5). Asia was the main origin of imported dengue fever (3,097 cases, 92.4%), chikungunya, VL, JE, and other diseases. Africa was the main region of origin for imported malaria (14,854 cases, 54.0%), and others, especially *Plasmodium falciparum*, yellow fever, loiasis, and other diseases (Table 3).

**Table 3:** Imported infectious diseases in mainland China, by region of origin, 2005–2016*

Disease	Africa	Asia	Oceania	Europe	North America	South America	Central America	Not ascertainable	Total
Malaria	14,854	9,160	68	5	2	18	0	3,390	27,497
Dengue	129	3,097	44	6	7	25	0	43	3,351
Influenza A(H1N1)	7	268	122	62	187	7	0	120	773
Chikungunya	2	12	0	0	0	1	0	3	18
Lyme disease	0	0	0	1	1	0	0	0	2
Scrub typhus	0	7	0	0	0	0	0	0	7
VL	1	12	0	1	0	0	0	0	14
JE	0	17	0	0	0	0	0	0	17
AFP	0	2	0	0	0	0	0	0	2
EHEC	0	3	0	0	0	0	0	0	3
EHF	5	2	0	0	0	0	0	0	7
Loiasis	11	0	0	0	0	0	0	0	11
Schistosomiasis	1	0	0	0	0	0	0	0	1
CCHF	1	0	0	0	0	0	0	0	1
MERS	0	1	0	0	0	0	0	0	1
Yellow fever	10	0	0	0	0	0	0	0	10
Zika virus	0	0	3	0	0	19	2	0	24
Total	15,021	12,581	237	75	197	70	2	3,556	31,740
%	47.3	39.6	0.8	0.2	0.6	0.2	0	11.2	100

*AFP, acute flaccid paralysis; CCHF, Crimean-Congo hemorrhagic fever; EHEC, *Escherichia coli* O157:H7; EHF, epidemic hemorrhagic fever; JE, Japanese encephalitis; MERS, Middle East respiratory syndrome; VL, visceral leishmaniasis.

Myanmar was the main country of origin for 5 imported diseases. These diseases were malaria (7,888 cases, 28.7%), dengue fever (1,384 cases, 41.3%), scrub typhus (6 cases, 85.7%), JE (13 cases, 76.5%), and acute flaccid paralysis (2 cases, 100.0%) (additional data not shown).

Malaria was imported mainly from Africa (14,854 cases, 54.0%) and Asia (9,160 cases, 33.3%). *P. vivax* came mainly from Asia, especially Myanmar (peaked in 2011), and was introduced into Yunnan Province during 2005–2016. There was an exception, however: Ghana exported more cases of *P. vivax* malaria to China in 2013. After that introduction, the number of imported cases of *P. vivax* malaria from Ethiopia, Angola, Equatorial Guinea, and other countries in Africa began to increase slightly. *P. falciparum* malaria also came mainly from Asia, especially from Myanmar, which exported it into Yunnan Province until 2013. In 2013, the largest number of cases of *P. falciparum* malaria came from Ghana; thereafter, most cases were imported from Angola, Nigeria, Equatorial Guinea, Ghana, and other countries in Africa, mainly into Guangxi, Jiangsu, and other southeastern provinces in China (data not shown).

Imported dengue fever cases were mainly from Asia, especially from Myanmar; most of them were brought into Yunnan and Guangdong Provinces. Influenza A(H1N1) was imported mainly from the United States (141 cases, 18.2%) and Australia (111 cases, 14.4%). The 1 case of MERS was imported from South Korea in 2015. All 11 imported cases of yellow fever came from Angola. Of the 24 imported Zika cases, 70.8% (17 cases) were from Venezuela.

## Discussion

Many infectious diseases, such as *P. vivax* malaria, dengue fever, influenza A(H1N1), epidemic hemorrhagic fever, JE, chikungunya, Lyme disease, scrub typhus, and VL, are found frequently in the indigenous population of China, but some are found more often as imported diseases, such as *P. falciparum* malaria, yellow fever, Zika virus, and MERS. Imported *P. falciparum* malaria is a major obstacle to achieving malaria elimination in China (*12**–**14*). This study showed that malaria was the most frequent imported infectious disease during 2005–2016, and Yunnan was the province with the greatest number of cases of imported malaria, which was consistent with other relevant studies in China (*15**–**17*). The reason for the large number of imported cases is that Yunnan Province has long international borders with Myanmar, Laos, Vietnam, and other countries of the Greater Mekong Subregion that show a high incidence of malaria (*16*). The persons who cross these borders to enter or leave China increase opportunities for infectious diseases to be imported from adjacent countries (*3**,**14**,**18**,**19*), especially from Myanmar (*12**,**13**,**20*).

*P. falciparum* malaria was imported mainly from Ghana and *P. vivax* malaria mainly from Asia, which was consistent with Zhou's findings (*5*). The number of cases of *P. falciparum* malaria imported into Guangxi Province increased significantly in 2013; this increase can be attributed mainly to cases imported from Ghana in 2013, which was related to people working in Ghana and other Africa countries during a gold rush in that year (*21**,**22*).

Africa and Asia were the main origins of imported malaria and other mosquitoborne diseases, findings consistent with those of Tian et al. (*23*). First, as a result of the rapid development of international economic exchange, trade, and travel, the number of migrant workers from China in Africa and Asia has increased annually in recent years. Second, climate and sanitary conditions in Africa and Southeast Asia are suitable for mosquitoes. Migrant workers from China in these areas are engaged mostly in outdoor field work, and their working and living environments are not mosquito preventive. Therefore, they have greater risk for infection (*24**,**25*). In addition, their health education level and self-protection awareness are low. All these factors have resulted in an increased number of malaria and other mosquitoborne diseases (*26*). Therefore, the main challenges of eliminating *P. falciparum* malaria are curtailing border malaria and imported cases from Myanmar and countries in Africa (*6**,**14*). Cooperation between China and neighboring countries has played an important role in improving malaria control at cross-border areas and should be further strengthened.

This study revealed that imported cases of infectious diseases such as malaria, dengue fever, and chikungunya were more common in male youths, which is consistent with the findings of Jiao et al. (*24*). Industries that use labor from China generally include construction, manufacturing, and transportation; therefore, the proportion of young men was high among migrant workers from China, to meet the needs of these industries.

Because of the distributions of mosquitoes and other vectors in China, diseases such as dengue fever, *P. vivax* malaria, JE, epidemic hemorrhagic fever, and chikungunya are acquired primarily locally. However, the numbers and the types of imported infectious diseases reported have increased in recent years. We indicate 4 main reasons for this increase. First, globalization is a major factor. With growing economic globalization, ongoing development of international trade, and convenient and fast transportation in recent years, factors such as microbial mutation, global warming, floods, droughts, and natural migration of animals as disease vectors have increased the risk of disease introduction into China (*27**–**29*). Second, these increases are related to the increases in the number of migrant workers from China, as well as increases in the number of tourists. This study showed that migrant workers from China accounted for a large proportion of imported malaria. Migrant workers and tourists are more at risk than local residents because of lack of preexisting immunity that dramatically increases the chances that they could become infected with these diseases and bring them in from abroad (*19*). Third, the level of surveillance, diagnosis, and detection of emerging or reemerging infectious diseases improved in China during the study years, which potentially contributed to the increase in the number and the types of cases with a confirmed diagnosis. Finally, some of the increases were caused by newly discovered or emerging infections, such as MERS, which was discovered in 2012 and confirmed in 2013; the 1 case imported into China was a result of the large outbreak in South Korea in 2015.

This study has analyzed the epidemiologic characteristics of all imported infectious diseases in mainland China over a recent 10-year period to provide scientific guidance for control and prevention of imported diseases in China. The study also has limitations. First, there is currently no special reporting system for imported infectious diseases in China. The imported cases that were analyzed in this study were reported through the National Information Reporting System of Infectious Diseases and identified by local CDC staff through epidemiologic investigation; therefore, not all imported cases may have been identified. A second limitation is that the data of this study were obtained from the monitoring system and there are few denominator data to use for calculating the rates of imported infectious diseases, so conclusions are limited and risks cannot be calculated. Finally, the factors concerning imported infectious diseases are complex, whereas our data are limited. Some demographic characteristics; trip information, such as purpose and duration of travel; and climate, environmental, and other relevant information could not be obtained. In subsequent research, we will focus on the collection of more information and continue to conduct our in-depth study of imported infectious diseases.

To control and prevent imported infectious diseases, we recommend several measures. First, pretravel education targeting infectious diseases that are endemic or prevalent in the destination countries is key for the prevention of imported diseases, especially imported malaria. Such education includes pretravel special training sessions; distribution of pamphlets or leaflets; and posttravel tips via notices, banners, or scrolling electronic screens at international entry and exit ports. Also important are dispensing of free preventive medications for malaria prophylaxis, self-treatment of severe travelers’ diarrhea, and vaccination against vaccine-preventable diseases such as JE and yellow fever during international travel and residence (*30*).

Second, border screening should be strengthened and improved. The effectiveness of border screening is controversial (*31*), but in recent years, China’s screening practices have proven to be a crucial measure for discovering imported infectious diseases in travelers who are ill at the time they cross a border. Screening has played a major role in prevention as the first line of defense against importation of foreign infectious diseases. For example, among 25 cases of Zika virus disease imported into mainland China in 2016, border screening recognized 9 (*11*), and of 11 cases of imported yellow fever reported in 2016, border screening recognized 6 (*32*). Border screening should be strengthened and improved to prevent those epidemic diseases and others from entering China (*32**–**34*).

Third, as a second line of defense against imported diseases, fever clinics and primary clinicians need to play an active role in identifying patients with imported infectious diseases. At present, many problems exist in fever clinics set up in medical institutions, such as failure to meet requirements, unqualified procedures, or nonstandardized management, and some locations may even be out of service. Clearer management standards are needed to provide a better chance for infectious disease screening. Training of medical staff, especially primary clinicians, should be reinforced to improve the ability to identify, diagnose, and treat emerging or reemerging infectious diseases and to ensure that imported cases can be diagnosed and control measures can be implemented as early as possible to prevent these diseases from further spreading in China.

Fourth, multisectoral and regional cooperation mechanisms, especially international cooperation mechanisms in the border areas, should be further enhanced (*35*). We suggest that the relevant departments should intensify cooperation by using well-defined responsibilities and should improve communication regarding all aspects of public information sharing, training, monitoring, and control. It is critical that, in the border areas, neighboring countries develop the management of persons entering and leaving, improve the control of mosquitoes, and jointly respond to infectious diseases.

Fifth, it is necessary to improve the early warning and response capacity for emerging infectious diseases. We recommend establishing a special system of surveillance, risk assessment, and early warning. Spatiotemporal models linking disease data and different environmental factors are also urgently needed (*36*).

In summary, our study found that the numbers of emerging infectious diseases imported into China have increased year by year. Therefore, we must pay closer attention to prevention and control of imported cases, while preventing and controlling indigenous cases. These factors are crucial for preventing and controlling infectious diseases, such as *P. falciparum* malaria, that have a large number of imported cases and seriously hinder the process of China eliminating malaria and other diseases.
